# Public release of hospital quality data for referral practices in Germany: results from a cluster-randomised controlled trial

**DOI:** 10.1186/s13561-017-0171-5

**Published:** 2017-09-26

**Authors:** Martin Emmert, Nina Meszmer, Lisa Jablonski, Lena Zinth, Oliver Schöffski, Fatemeh Taheri-Zadeh

**Affiliations:** 1 School of Business and Economics, Health Services Management, University of Erlangen-Nuremberg, Lange Gasse 20 90403 Nuremberg, Nuremberg, Germany; 2 Media, Information and Design Department of Information and Communication, University of Applied Sciences and Arts, Hannover, Germany

**Keywords:** Public reporting, Hospital quality, Patient referral, Patient counselling, Public health

## Abstract

**Objective:**

To evaluate the impact of different dissemination channels on the awareness and usage of hospital performance reports among referring physicians, as well as the usefulness of such reports from the referring physicians’ perspective.

**Data sources/Study setting:**

Primary data collected from a survey with 277 referring physicians (response rate = 26.2%) in Nuremberg, Germany (03–06/2016).

**Study design:**

Cluster-randomised controlled trial at the practice level. Physician practices were randomly assigned to one of two conditions: (1) physicians in the control arm could become aware of the performance reports via mass media channels (Mass Media, ${n}_{MM}^{pr}$=132, ${n}_{MM}^{ph}$=147); (2) physicians in the intervention arm also received a printed version of the report via mail (Mass and Special Media, ${n}_{MSM}^{pr}$=117; ${n}_{MSM}^{ph}$=130).

**Principal findings:**

Overall, 68% of respondents recalled hospital performance reports and 21% used them for referral decisions. Physicians from the Mass and Special Media group were more likely to be aware of the performance reports (OR 4.16; 95% CI 2.16–8.00, *p* < .001) but not more likely to be influenced when referring patients into hospitals (OR 1.73; 95% CI 0.72–4.12, *p* > .05). On a 1 (very good) to 6 (insufficient) scale, the usefulness of the performance reports was rated 3.67 (±1.40). Aggregated presentation formats were rated more helpful than detailed hospital quality information.

**Conclusions:**

Hospital quality reports have limited impact on referral practices. To increase the latter, concerns raised by referring physicians must be given more weight. Those principally refer to the underlying data, the design of the reports, and the lack of important information.

**Electronic supplementary material:**

The online version of this article (doi: 10.1186/s13561-017-0171-5) contains supplementary material, which is available to authorized users.

## Background

The aim of public reporting is to improve healthcare quality by both stimulating quality improvement on the provider level (“Improvement Through Changes in Care”) and also by helping patients and other consumers select the “right” provider (“Improvement Through Selection”) [1]. While the published literature has confirmed the potential for public reporting to induce changes in clinical practice [2–5], little to no impact on the selection of healthcare providers has been demonstrated [2, 3]. So far, most of the literature has addressed whether patients use publicly reported quality information to search for and to select health care providers [6–10]. However, less information is available regarding whether public reporting plays a role from the physicians’ perspectives for referring patients to hospitals.

The available international literature suggests a limited impact of publicly reported quality information on the hospital referral behavior of physicians. For example, surveys of cardiologists in Pennsylvania in 1996 [11] and New York in 1997 [12] have revealed that even though most cardiologists were aware of cardiac surgery report cards, the impact of these on their hospital referral behavior was limited. Two decades later, despite an almost universal awareness of cardiac surgeon report cards, their impact on referral practices still remains limited [13]. Also, recent evidence from France [14] and the Netherlands [15] backs up those findings. The issue of whether and how physicians use publicly reported hospital quality information for referring patients to hospitals in Germany has yet to be addressed. In addition, there is still a gap in international research in evaluating the effectiveness of different dissemination channels [16] and presentation formats [17, 18] to maximize the impact of performance reports. Therefore, this study explores the impact of different dissemination channels on the awareness and usage of a hospital performance reporting initiative, namely the *Nuremberg hospital quality reporting system (NHQRS)*, among referring physicians, as well as the usefulness of such reports from the referring physicians’ perspective. The latter publicly reported about the quality of care of hospitals for 14 clinical procedures in the region of Nuremberg (Bavaria, Germany) between January and April 2016.

### New contribution

To the best of our knowledge, no evidence regarding the impact of public reporting on hospital referrals in the German healthcare setting has been published. Some survey results show that quality performance information does not play an important role for hospital referrals [19–21] but no study has yet addressed the impact of a public reporting intervention on referral behavior. When looking at this from an international perspective, research is necessary to evaluate the effectiveness of different dissemination channels in order to maximize the impact of performance reports [16]. In this context, Gombeski and colleagues have developed a model that illustrates the interaction of factors and channels influencing the referral decision. It shows that information about a referral organization/physician flows to physicians through interpersonal media (e.g., met physician at events, social event), mass media (e.g., television, newspapers), or special media (e.g., direct mail, brochures) [22]. We therefore hypothesize that a combination of such dissemination channels will increase awareness compared with a single approach. Barr and colleagues have further raised the question whether greater awareness of public reports will result in more willingness by physicians to use these reports in decision making about patient care [16]. Based on this, we investigate whether an increased level of awareness will also result in a greater willingness to use the NHQRS for referring patients to hospitals. Finally, studies have shown that less complex information displays should be employed to increase the comprehensibility and usage of report cards [17, 23]. So far, most research has focused on patients as the target group of report cards. Given evidence from a recently published systematic review, no research has addressed whether physicians also prefer simplified information displays for hospital referrals [17]. We thus evaluate the usefulness of different quality presentation formats (e.g., ordering providers, presenting composite measures, different amounts of quality information) from the physicians’ perspective for referral practices.

As follows, three major research questions guided this study:

1. To what extent do physicians become aware and make use of publicly reported hospital quality reports when referring patients to hospitals?
2. Does a combined mass and special media dissemination approach (i.e., newspaper and direct mailing) lead to higher awareness and usage results compared with a single mass media dissemination approach (i.e., newspaper)?
3. What are physicians’ attitudes and perceptions of the performance reports in general and of different presentation formats in particular?

## Methods

### The Nuremberg hospital quality reporting system (NHQRS)

The NHQRS was developed by the authors of this investigation in cooperation with a local newspaper (Nürnberger Zeitung) with the aim of publicly reporting about the quality of hospitals in a region 50 km around Nuremberg, located in the south of Germany. In 2016, the estimated population for Nuremberg, where the Nürnberger Zeitung is mainly distributed, was estimated to be 529,047. Quality results were published on a weekly basis every Saturday in the local newspaper (Nürnberger Zeitung; daily print circulation approx. 2016: 33,000) and on corresponding online media websites (e.g., Facebook, nordbayern) between January 2nd and April 9th 2016. [Number of fans of the Facebook page of the Nürnberger Zeitung 01/2016: 53,047 fans; Number of followers on Twitter 12/2016: 59,740.] The public reporting intervention encompassed quality results for 14 clinical procedures, such as gall-bladder resection, artificial hip replacement, and percutaneous coronary intervention. Those procedures were chosen based on the availability of clinical performance data as well as on the number of cases in Germany.

Four different *data sources* were used to determine hospital quality: (1) The German external quality assurance: hospital treatment for selected interventions is documented for each patient based on a set of in-house related quality indicators. Currently, the assurance system comprises 400 quality indicators within 30 different clinical areas [24]. Besides other objectives, these data can be used for public reporting purposes [19, 23, 25]; [For the special purpose of the NHQRS, all available publicly reported quality indicators with a defined reference range were used.] (2) Insurance claims data (Allgemeine Ortskrankenkasse): these data allow for an assessment of hospital quality based on routine data which enable a long-term perspective after hospital discharge (e.g., up to 365 days in prostate cancer treatment) [26]. [Here, all publicly reported quality indicators were used; see [26] for further statistical information regarding the underlying ranking procedure.] (3) The number of cases treated in 2014, as well as (4) patient satisfaction. The latter was derived from a patient satisfaction survey administered by two German statutory health insurances (AOK, BARMER GEK) and one hospital report card provider (the Weisse Liste). So far, roughly 2,000,000 patients have answered the Patients’ Experience Questionnaire (PEQ) [27, 28].

Based on those data, we developed *three different presentation formats* to display different levels of hospital quality information. For example, a patient might be less interested in very detailed quality information in contrast to referring physicians or hospital management. Of these, two versions were published in the newspaper. They included one short overall ranking, whereby all hospitals were ranked into 3–5 performance groups without presenting any quality information (Version 1), as well as one alphabetical overview showing a composite score for each of the four data sources (Version 2) (see above). For developing version 1, we assigned a higher weight to medical quality and assigned hospitals to 3–5 different performance groups according to medical quality results (i.e., based on the the German external quality assurance data and insurance claims data). For example, hospitals with better than average results in both analysis were assigned to the first performance group. In constrast, those with lower than average results were assigned to the last performance group. Afterwards, the ordering within each performance group was based on the number of cases (i.e., below average, average, above average) and patient satisfaction scores. Besides this, one detailed presentation format was published online showing the results for each hospital on a quality-indicator based level (Version 3) (See Additional file 1).

### Study design

Our study was designed as a cluster-randomized controlled trial at the physician practice level. Based on previously published literature discussing different information dissemination models [16, 22], we randomly assigned referring physicians (*n* ^*pr*^=789, *n* ^*ph*^=1057; where *n* ^*pr*^, *n* ^*ph*^ denote the total number of practices and physicians in the region 25 km around Nuremberg, respectively) either to the control group (“Mass Media”; ${n}_{MM}^{pr}$=381, ${n}_{MM}^{ph}$=527) or the intervention group (“Mass and Special Media”; *n*=408, ${n}_{MSM}^{ph}$=530) using a software-generated random number table. While mass media are defined as channels of communication aimed at the public with no opportunity for immediate feedback (e.g., newspapers, television, radio), special media are defined as highly focused communication channels designed for specific audiences with no immediate feedback (e.g., direct mail, brochures, books) [22]. Thus, physicians from the Mass Media group did not receive any additional information but could become aware of the NHQRS via the newspaper or other mass media channels. In contrast, physicians from the Mass and Special Media group also received a printed version of the newspaper article via mail.

We surveyed all referring physicians in the region 25 km around Nuremberg between March and June 2016. Thereby, we contacted referring physicians from six clinical areas 2 months after the publication of the results for the relevant clinical procedure. The physicians included both general practitioners and specialists (i.e., orthopedists, gastroenterologists, urologists, gynecologists, cardiologists). In a first step, physicians with an online available email address were contacted via email which contained a link to participate online (in a web-based survey), while the remaining physicians were contacted via regular mail and received a printed version of the survey (see Additional file 2 for the survey instrument). The cover letter contained some information about the NHQRS, the study and its purpose. After 1 week, a first reminder was sent out, followed by a second reminder a week later. The questionnaire was piloted by 25 individuals to ensure the comprehensibility of the wording and internal validity; final adjustments were made accordingly. The online survey was designed using Questback’s internet-based EFS survey software and was also pre-tested. As an incentive, we held draws for one of four Amazon vouchers with a value of €150 each.

### Data analysis

The applied survey contained both scaled-survey questions as well as open-ended text questions. We thus used a mixed-method approach by analyzing quantitative as well as qualitative data [29]. Quantitative results are presented as both means and standard deviations for continuous variables and as numbers and percentages for categorical variables. We performed comparisons between two groups by using a Chi-square test (two-sided) for categorical variables and a Mann-Whitney U test for continuous nonparametric variables. In addition, generalized estimating equation (GEE) models were performed to identify the main predictors associated with awareness and usage of the NHQRS by considering demographic (age, gender), professional (medical discipline, practice type, performing the publicly reported procedure), and study-related (e.g., Mass Media vs. Mass and Special Media group) characteristics. The generalized estimating equations are used for analyzing data from clustered randomized controlled trials on the individual level by accounting for the structure of the correlations within clusters [30–32]. The structure of GEE is like generalized linear models (GLM) defined by

$$ \left(E(Y)\right)={\beta}_0+{\beta}_1{X}_1+{\beta}_2{X}_2+\cdots +{\beta}_k{X}_k $$

where *Y* denotes the response variables awareness respectively usage and *X*_1_ , *X*_2_ ,  ⋯  , *X*_*k*_ denote the independent variables.

Due to the binary form of the response variable *Y* (i.e., awareness/usage), we applied the logit function $g(p)=\mathit{\log}\left(\frac{p}{1-p}\right)$as the link.

The QIC- and QICC-statistic were applied as the goodness-of-fit and model-selection criterion for our GEE based models [33]. All statistical analyses were conducted using SPSS version 23.0 (IBM Corp. Released 2012. IBM SPSS Statistics for Windows, Armonk, NY: IBM Corp.). Observed differences were identified as statistically significant if *p* < .05, and highly significant if *p* < .001.

Besides, qualitative analysis techniques were used to evaluate all open-ended text comments. We created a posteriori codebook by using small sets of between five and 10 reviews. Two evaluators coded independently and discussed the discrepancies. The codebook was updated in an iterative process until no new codes were identified. Some main categories were split into major and minor themes based on a directed qualitative content analysis method [34–36]. Finally, qualitative results were converted into quantitative data [37]. To ensure accuracy of coding, one author checked the qualitative approach by second-level coding.

## Results

Our final study sample consisted of 277 respondents who completed the survey (overall response rate = 26.2%). The mean age was 54.67 (±8.47) years, 31.8% of the respondents were female and slightly more than half of the respondents were specialists (54.5%) (Table 1). The Mass Media group consisted of 147 physicians (response rate = 27.9%; ${n}_{MM}^{pr}$=132, ${n}_{MM}^{ph}$= 147) and the Mass and Special Media group was comprised of 130 physicians (response rate = 24.5%; ${n}_{MSM}^{pr}$= 117; ${n}_{MSM}^{ph}$= 130), respectively. As shown, no statistically significant differences between both study groups in terms of age, gender, medical discipline, and practice type (*p* > .05 each) could be detected, thereby demonstrating an effective randomization process.

**Table 1:** Overview of the study sample (*p* value was calculated using chi-square test)

Characteristics	Study sample (*n* ^*ph*^=277)	Mass media (${n}_{MM}^{ph}$=147)	Mass and special media (${n}_{MSM}^{ph}$=130)	p
Age				
18 to 44 years	12.3%	16.0%	8.5%	.358
45 to 49 years	16.1%	16.0%	16.2%	
50 to 54 years	19.1%	18.5%	19.7%	
55 to 59 years	22.0%	22.7%	21.4%	
60 to 64 years	16.9%	12.6%	21.4%	
65 years and older	13.6%	14.3%	12.8%	
Gender				
Male	68.2%	67.3%	69.2%	.737
Female	31.8%	32.7%	30.8%	
Medical discipline				
General practitioner	45.5%	44.9%	46.2%	.834
Specialist	54.5%	55.1%	53.8%	
Practice type				
Single physician practice	43.0%	45.2%	40.7%	.747
Multiple physician practice	45.0%	42.7%	47.5%	
Medical care unit	12.0%	12.1%	11.9%	

### Study design

Cluster-randomised controlled trial at the practice level. Physician practices were randomly assigned to one of two conditions: (1) physicians in the control arm could become aware of the performance reports via mass media channels; (2) physicians in the intervention arm also received a printed version of the report via mail.

### Awareness of the NHQRS

Awareness of the NHQRS was reported by 177 (68.3%) respondents. As presented in Table 2, there were no statistically significant differences regarding age, gender, medical discipline, whether or not performing the publicly reported procedure, the communication measure as well as the time of the survey (*p* > .05 each). In contrast, physicians who work in any form of multiple physician practices (74.8% vs. 58.7%, *p* < .05), or those from the Mass and Special Media group were shown to have significantly higher awareness levels of the NHQRS (83.2% vs. 54.5%, *p* < .001).

**Table 2:** Descriptive analysis and GEE based regression model predicting awareness of NHQRS

Parameter	Description (Overall 68.3%)	Regression analysis					
		Regression coefficient	Standard error	Odds ratio	95%-Wald CI lower bound	95%-Wald CI upper bound	*p*-value
Age		−0.020	0.105	0.98	0.80	1.21	0.851
Gender							
Male	71.3%						
Female	62.4%	−0.312	0.341	0.73	0.38	1.43	0.359
Medical discipline							
General practitioner	63.6%						
Specialist	72.3%	0.613	0.788	1.85	0.39	8.65	0.437
Practice type							
Single physician	58.7%*						
Practice otherwise	74.8%	0.474	0.332	1.61	0.84	3.08	0.153
Performing publicly reported procedure							
No	69.2%						
Yes	69.7%	−0.316	0.468	0.73	0.29	1.82	0.500
Dissemination channel							
Mass Media	54.5%**						
Mass and Special Media	83.2%	1.425	0.334	4.16	2.16	8.00	<0.001
Communication measure (survey)							
Email	69.8%						
Mail	67.6%	−0.341	0.368	0.71	0.35	1.46	0.354
Time of survey							
Before the last published report	63.6%						
After the last published report	72.3%	−0.208	0.803	0.81	0.17	3.92	0.795
Constant	–	0.244	0.547	1.28	0.44	3.73	0.655

The first expression of the variable is regarded as the reference group

Age considered as a continuous variable in the model

QIC = 259.68, QICC = 260.07

*p < 0.05 (using chi-square test)

**p < 0.001 (using chi-square test)

The regression model based on generalized estimating equations on the physician level showed that demographic and professional characteristics were not associated with awareness of the NHQRS. However, the dissemination channel of the quality information was shown to be significantly associated with awareness of the reporting system; the odds of being aware of the performance reports is 4.2 times greater for physicians from the Mass and Special Media group compared with those from the Mass Media group, all other variables of the model being held constant (OR 4.16; 95% CI 2.16–8.00, *p* < .001).

### Impact of the NHQRS on referral practices

Overall, every fifth physician (20.6%) stated that he/she had been influenced by the NHQRS when referring patients to hospitals (Table 3). The results indicate no statistically significant differences regarding age, gender, practice type, whether or not performing the publicly reported procedure, and the communication measure of the survey (*p* > .05 each). In contrast, general practitioner (33.3% vs. 10.8%, *p* < .001), those who were surveyed after the last published report (31.5% vs. 9.1%, *p* < .05), or those from the Mass and Special Media group were shown to have significantly higher impact levels of the NHQRS (26.0% vs. 13.2%, *p* < .05). More specifically, almost every sixth physician stated that NHQRS had had an impact either in a positive or negative direction (15.0% vs. 14.9%).

**Table 3:** Descriptive analysis and GEE based regression model predicting impact of NHQRS on hospital referrals

Parameter	Description (Overall 20.6%)	Regression analysis					
		Regression coefficient	Standard error	Odds ratio	95%-Wald CI lower bound	95%-Wald CI upper bound	*p*-value
Age		−0.184	0.142	0.83	0.63	1.77	0.196
Gender							
Male	17.7%						
Female	26.8%	0.072	0.429	1.08	0.46	2.49	0.866
Medical discipline							
General practitioner	33.3%**						
Specialist	10.8%	−0.721	0.899	0.49	0.08	2.83	0.422
Practice type							
Single physician practice	19.1%						
Otherwise	20.6%	0.339	0.431	1.40	0.60	3.27	0.431
Performing publicly reported procedure							
No	24.2%						
Yes	11.5%	0.048	0.734	1.05	0.25	4.42	0.948
Dissemination channel							
Mass Media	13.2%*						
Mass and Special Media	26.0%	0.546	0.443	1.73	0.72	4.12	0.219
Communication measure (survey)							
Email	15.3%						
Mail	23.1%	0.684	0.515	1.98	0.72	5.43	0.184
Time of survey							
Before the last published report	31.5%*						
After the last published report	9.1%	−1.155	0.880	0.32	0.06	1.10	0.189
Constant	–	−1.216	0.712	0.296	0.07	1.20	0.088

The first expression of the variable is regarded as the reference group

Age considered as a continuous variable in the model

QIC = 158.83, QICC = 158.98

*p < 0.05 (using chi-square test)

**p < 0.001 (using chi-square test)

The regression results could not reveal any demographic and professional characteristics to be associated with impact of the NHQRS on hospital referrals. Here, the dissemination channel of the quality information could not be shown to be a significant predictor of impact on hospital referrals (OR 1.73; 95% CI 0.72–4.12, *p* > .05).

Based on a paper of Brown and colleagues [13], we performed another regression model without considering the time of the survey (i.e., before or after the last published report). In line with the findings above, the dissemination channel of the quality information could not be shown to be a significant predictor of impact on hospital referrals (OR 1.88; 95% CI 0.80–4.39, *p* > .05). However, we could detect significantly lower odds for specialists compared with general practitioner (OR 0.20; 95% CI 0.07–0.58, *p* < .05).

### The usefulness of the NHQRS

Based on the German school grading system (1 = very good to 6 = insufficient), the NHQRS was rated 3.67 (±1.40) (Table 4). More specifically, an assessment of the trustworthiness, helpfulness, credibility, and informative value indicates slightly less favorable results in these areas. On a 1 (not trustworthy etc. at all) to 5 (very trustworthy etc.) scale, the results vary between 2.49 (±1.16) for helpfulness and 2.85 (±1.08) for credibility, respectively. Therefore, the more detailed versions of the reporting formats were rated as less helpful (version 1: 2.69 ± 1.34; version 2: 2.43 ± 1.24; version 3: 2.35 ± 1.27). The future impact on referral practices was shown to be highest for the overall ranking result (2.29 ± 1.33), and lowest for the second version that presented hospitals in alphabetical order along with the overall performance scores for all four data sources (2.11 ± 1.18), respectively. It is interesting to note that general practitioners gave significantly better ratings than specialists regarding all issues (*p* < .05 each). We also analyzed rating results between both study groups according to the dissemination channel of the information but did not detect any significant differences (not presented here).

**Table 4:** The usefulness of the NHQRS (*p* value was calculated using Mann-Whitney U)

Rating	Overall (*n* ^*ph*^=277)	General practitioner (*n* ^*ph*^=126)	Specialist (*n* ^*ph*^=151)	p
Overall rating^a^	3.67 (1.40)	3.37 (1.37)	3.92 (1.38)	.002
Detailed rating categories^b^				
Trustworthiness^b^	2.68 (1.08)	2.89 (0.99)	2.50 (1.12)	.003
Helpfulness^b^	2.49 (1.16)	2.77 (1.15)	2.25 (1.12)	<.001
Credibility^b^	2.85 (1.08)	3.03 (1.03)	2.69 (1.10)	.009
Informative value^b^	2.65 (1.14)	2.87 (1.14)	2.47 (1.11)	.004
Version 1 (overall ranking, 3–5 performance groups)				
Helpfulness^b^	2.69 (1.34)	3.05 (1.30)	2.38 (1.29)	<.001
Likely impact on future referral practice^b^	2.29 (1.33)	2.66 (1.31)	1.98 (1.26)	<.001
Version 2 (alphabetical order; categorical overall performance)				
Helpfulness^b^	2.43 (1.24)	2.66 (1.21)	2.23 (1.23)	.004
Likely impact on future referral practice^b^	2.11 (1.18)	2.38 (1.19)	1.88 (1.13)	<.001
Version 3 (alphabetical order; detailed quality indicator-based information)				
Helpfulness^b^	2.35 (1.27)	2.64 (1.30)	2.10 (1.20)	.001
Likely impact on future referral practice^b^	2.22 (1.25)	2.56 (1.29)	1.92 (1.13)	<.001

^a^German school-based rating system; 1 to 6 scale (1 = very good; 2 = good; 3 = satisfactory; 4 = sufficient; 5 = deficient; 6 = insufficient)

^b^1 to 5 scale (1 = not trustworthy etc. at all; 5 = very trustworthy etc.)

### Critical analysis of the NHQRS

In total, 68 open-ended text answers were analyzed which comprised 147 critical statements about the usefulness of the NHQRS (Table 5). Most frequently, referring physicians criticized the underlying data (*n* = 38), particularly with respect to the appropriateness of the data used concerning claims (*n* = 9) and the risk of manipulation of the data (*n* = 6). Furthermore, 21 comments were related to the design of the ranking, such as the placement of explanatory information (*n* = 6), type size (*n* = 3), order of hospitals (*n* = 2) or the traffic light color-based design used in version 1 (*n* = 2). Twenty comments contained statements that important quality information was missing. For example, urologists stated that aspects such as mortality or complications within 30 days after surgery were included, but not relevant patient-reported outcome measures (e.g., continence success, potency success). Furthermore, others raised the importance of integrating the satisfaction of the referring physicians.

**Table 5:** Results of the qualitative analysis regarding criticism from the physicians’ perspective

Topic of the criticism	N	Examples
Flaws of underlying data (e.g., timeliness, risk-adjustment, validity, risk of manipulation)	38	• “The number of cases based on insurance claims data is not sufficiently large” • “Individual data are too often manipulated”
Design (e.g., type size, placement of information, rank order, traffic light colors)	21	• “The alphabetical order is not helpful” • “Tables with insurance claims data include terms far too complex”
Missing of further quality information (e.g., PROMs, satisfaction of referring physicians)	20	• “Access to hospitals is not included” • “You should survey referring physicians”
Impact of the public reporting initiative (e.g., risk of misinformation, short-term impact)	11	• “Experience shows that such actions cause uncertainty for patients” • “The effect is of limited duration”
Methodology for deriving the quality scores (e.g., weighting of quality information)	10	• “The methodical approach to deal with this issue does not reflect reality” • “The data were not assessed accurately”
Publication media (e.g., daily newspaper inappropriate)	10	• “I do not use quality information provided by a newspaper” • “I believe that the newspaper NZ is not the appropriate media to publish scientific data”
Other factors more relevant (e.g., own experience, patient preference, distance)	10	• “I rely on results which I can see on patients and patients’ experiences myself” • “Personal experiences are more important”
Hospital related issues (e.g., ownership structure)	8	• “No consideration of multi-morbid patients, which are treated in small hospitals - > bad grade of large hospitals” • “Regarding the fact that “private hospitals” treat very highly selected patients (e.g. no risk patients), it is difficult to compare the number of cases and statistical analysis regarding mortality with local community hospitals”
Transparency (e.g., funding, methodology, conflict of interests)	5	• “Was there any relationship with the newspaper? (conflict of interests)?” • “I don’t know about the significance of the criteria, the transparency is questionable”
Ranking does not match physicians’ experience	4	• “Based on daily practice some hospitals are overrated” • “I was treated in hospitals which are rated as under-performer and I was extremely satisfied”
Structural changes in hospitals	3	• “Structures are changing quickly” • “Since 2013/2014 several surgeons have been changing hospitals (retirement, other hospital!)”
Scope of the public reporting initiative	3	• “A smaller number of hospitals would be easier to recognize” • “I cautiously read the Focus hospital ranking which contains also the remaining hospitals in Germany”
Subjectivity of the ranking	2	• “In my opinion such rankings are usually created by persons who have no or less insight in daily routines of a hospital. As a result subjective opinions/impressions are included” • “Shouldn’t there be more newspapers or institutes to conduct such a ranking?”
No specific reasons stated	2	• “Rankings seem generally suspicious to me” • “Rankings are very meaningful for the majority of the population. Whether rankings present the exact condition of hospitals is questionable”

## Discussion

Our results show that by publishing hospital quality information only in the mass media, almost six out of 10 physicians (55%) became aware of the NHQRS; this is mainly in line with findings from the US. For example, Schneider and Epstein showed that after publishing the Consumer Guide to Coronary Artery Bypass Graft (CABG) Surgery in Pennsylvania four times, 82% of cardiologists surveyed were aware of the data [11]. Two decades later, an almost universal awareness among cardiologists had been reached (94%) [13]. It is important to mention that the era of public reporting in Germany has just begun to develop. Even though the German Federal Joint Committee states that “To date, no other country in the world has a similar, nationwide procedure requiring documentation and online disclosure of health care quality performance in inpatient settings” [38], the awareness of such quality information still remains low in Germany. A survey showed that only 39% of physicians are aware of corresponding hospital quality reports and 11% of internet websites on which the information is publicly disclosed [21]. In contrast to previous evidence, we did not find any statistically significant differences regarding age [11]. We observed, however, significantly higher awareness among those who work in any form of multiple physician practices or those from the Mass and Special Media group.

We were able to show that the dissemination channel of the quality information matters. By sending a printed copy of the article via mail to the physicians’ practices we were able to significantly increase the awareness level by almost 30 percentage points. This has major implications for health policymakers with respect to achieving a rapid and broad awareness of hospital quality information among physicians. As mentioned above, the Consumer Guide to CABG surgery had been published four times in Pennsylvania starting in 1992 before Schneider and Epstein determined the awareness level to be 82% [11]. We could slightly exceed this awareness level in the first year of the NHQRS (here, 83.2% of physicians reported awareness) by combining mass and special media dissemination channels.

Every fifth physician (20.5%) stated that he/she had been influenced by the NHQRS when referring patients to hospitals, which is in line with international evidence. For example, in the study conducted by Schneider and Epstein, only 13% of the cardiologists surveyed responded that the Consumer Guide ratings had a moderate or substantial impact on their referral [11]. Hannan et al. showed slightly higher impact scores; in their study, 6% of the cardiologists surveyed responded that the New York CABG surgery reports had affected their referrals to surgeons “very much”, and 32% “somewhat”. In addition, only 22% stated that they routinely discussed the information in the cardiac report with their patients [12]. Two decades later, Brown et al. surveyed cardiologists in New York and showed that still, only 25% reported that the reports had a moderate or substantial influence on referral decisions, and 29% stated that they had discussed the report cards with patients [13]. Available studies from Europe have also demonstrated the limited impact of public reporting on referral behavior. For example, Ferrua et al. surveyed 503 self-employed general practitioners in private practice in France. They showed that approximately 14% of the practitioners had already used publicly available comparative indicators to influence hospital choices for their patients [14]. Similar results have also been shown in the Netherlands. For example, in a study by Ketelaar et al. only 12% of physicians surveyed reported that they had used comparative performance information when selecting a hospital [15].

It furthermore seems that the positive and negative impact of hospital performance reports are alike. In our study, almost every sixth physician stated that performance reports had had an impact either in a positive (15.0%) or negative (14.9%) direction. This confirms evidence from the US showing that 35% of physician rating website users reported selecting a physician based on good ratings, while 37% had avoided a physician with bad ratings. Slightly in contrast, two studies from Germany showed higher impact results in a positive direction for both hospitals [39] and physicians [9]. The higher impact results might be due to different survey samples. In contrast to our study, in which health care providers were surveyed, the mentioned studies have focused on the general population. Barr and colleagues have further raised the question concerning whether a greater awareness of public reports will result in more willingness by physicians to use those reports [16]. Based on our findings, the impact among physicians from the Mass and Special Media group, for whom statistically greater awareness levels were presented, could be shown to be significantly greater. However, the regression results could not prove the dissemination channel of the quality information to be a significant predictor of impact on hospital referrals.

Overall, the physicians surveyed gave a slightly less favorable rating regarding the value of the performance reports for making hospital referrals (mean 3.67 on a 1 = very good to 6 = insufficient scale), thus confirming international findings. For example, Hannan et al. surveyed 450 cardiologists in New York to determine the value of the CABG surgery outcomes for all hospitals in New York. Here, the average rating was 2.40 on a 1 (not at all useful) to 5 (extremely useful) scale. In addition, 84% of the respondents rated the report to be between “not at all useful” and “somewhat useful” [12]. We also found that general practitioners gave significantly more favorable ratings than specialists. Specialists probably do not feel the need for such information due to their more focused clinical areas. These individuals might feel more capable of overseeing possible hospitals for inpatient treatment while assessing the quality of them. In this regard, Epstein examined referral patterns to cardiac surgeons to assess whether public reporting added information to what referring physicians already knew [40]. As a result, he showed that CABG patients were significantly less likely to be treated by high-mortality surgeons and more likely to be treated by low-mortality surgeons even without the report cards. He concludes that referring specialists appear to have been knowledgeable about the relative performance of cardiac surgeons on their own without the need to use report cards. However, this finding is true for a medical intervention that is typically cared for by specialists and not general practitioners, who deal with a broader range of patients and diseases. General practitioners might need knowledge about treatment options for a greater variety of clinical areas, and thus they might see a greater benefit in publicly reported quality data.

Previous evidence has demonstrated that less complex information displays should be favored to increase the comprehensibility and usage of performance report cards among patients and other consumers [17, 23]. We initially hypothesized that this might not be true for referring physicians who are more likely to be interested in detailed hospital quality information. However, we determined that the most aggregated presentation format (overall composite measure based ranking, 3–5 performance groups) was rated the most helpful for referral decisions. This seems to be somewhat surprising; especially since only every fourth physician surveyed (27.3%) rated this presentation format as confidential. On the other hand, every second physician (50.5%) found it useful to present hospitals in different performance groups as we did. In contrast to other quality reporting initiatives, which publicly report only top-performing hospitals (e.g., The US 100 top hospitals, the US News hospital ranking, the German FOCUS Ärzteliste), we decided to present both high- and low-performing hospitals what was assessed as positive by approximately 70% of all physicians. Again, the presentation of hospital quality information on a quality-indicator based level in alphabetical order was rated as least helpful. Interestingly, only one of three physicians (34.5%) thought that such a detailed level was necessary to assess the quality of hospitals. Therefore, those who thought it was necessary gave a more favorable rating that those who did not (3.19 vs. 1.82; *p* < .001). Consequently, our findings supplement the results from a recently published systematic review [17] by demonstrating that the majority of referring physicians also seem to prefer simplified information displays.

Given these findings and the effort which has been put into further developing report cards in health care over the last two decades [41, 42], one might contemplate the reasons for the still limited impact. One major hurdle seems to be that physicians do not trust the publicly reported quality information and thus do not use it [12, 16, 43, 44]. Despite the incorporation of the best available hospital quality data in Germany, referring physicians in our study raised several concerns which need to be addressed. In line with international evidence, publicly reported data in Germany do not seem to include all relevant quality information [11, 13]. Exclusively focusing on clinical metrics (e.g., mortality or complications), the number of cases, or patient satisfaction does not suffice when making referral decisions. But, patient-reported outcome measures, which provide information that is relevant from a patient’s perspective, were missing. For example, in prostate cancer treatment, aspects such as continence and potency success are also important for patients undergoing prostatectomy surgery. Others wish further information such as the satisfaction of referring physicians or the case mix of the patients in each hospital. Others mentioned that some included quality metrics were even inappropriate to determine the quality of hospitals [13], and thus would be misleading for patients who are searching for a hospital.

There are some limitations that have to be taken into account when interpreting the results of our study. First, we achieved a response rate of 26%. This means that we cannot ensure representativeness for all referring practitioners in the region (nonresponse bias). Nevertheless, our response rate is within the range of studies with a similar approach [13, 45, 46]. Besides, literature has suggested that there is no consistent relationship between nonresponse rates and nonresponse bias [47]. Second, our results focus on the short-term impact of public reporting. We estimated the results regarding the awareness, usage, and impact 2 months after the public reporting intervention. Thus, we were not able to determine the long-term effect of the intervention as reported in other studies [11–13]. Furthermore, the findings about the impact of the NHQRS were calculated based on the responses of the surveyed referring physicians. Those results might differ from studies applying an experimental design under real conditions when analyzing empirical data regarding the impact, such as the numbers of cases per year. Finally, hospital quality information was published mainly in one regional newspaper. The publication in further newspapers might have led to higher awareness results.

## Conclusions

Despite all the efforts that have been undertaken to further develop public reporting [41, 42], its impact on hospital referrals is still limited. Based on our results, much has to be done if we want quality reporting initiatives to be more meaningful from the referring physician’s perspective. One the one hand, this is partly due to the low awareness levels concerning publicly available hospital quality information. In this regard, the dissemination channel of the quality information matters; for a rapid and broad awareness to be reached, a singular mass media approach does not seem to be appropriate. On the other hand, we assume that the limited impact of hospital quality reports is much more likely due to the fact that referring physicians do not trust the published information and thus do not make use of it [44].

### Implications for health policymakers

Before putting even greater effort into promoting publicly available quality information, health policymakers should rather address the concerns raised by referring physicians. These concerns mainly refer to different issues regarding the underlying data, the design of reporting initiatives, and the lack of important quality information. As stated by Mukamel and colleagues, “Quality report cards are only as good as the measures they include” [10]. Otherwise, many resources will be spent without significantly increasing the impact and benefit of reporting systems.

## Additional files

Additional file 1: An overview of the publicly reported Nuremberg Hospital Quality Reporting System (NHQRS). (PDF 1008 kb)

Additional file 2: The survey instrument. (DOCX 623 kb)
