# Supplementary material for: Public release of hospital quality data for referral practices in Germany: results from a cluster-randomised controlled trial
Source: Health Econ Rev. 2017 Sep 26;7:33. doi: 10.1186/s13561-017-0171-5 (PMC5615085; doi:10.1186/s13561-017-0171-5)
Supplement: Supplementary file 1 — An overview of the publicly reported Nuremberg Hospital Quality Reporting System (NHQRS). (PDF 1008 kb) [file 13561_2017_171_MOESM1_ESM.pdf]

Local clinic ranking

Gallbladder Removal

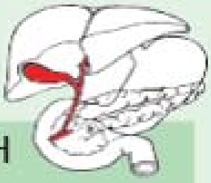

Rank	Clinics
1	Waldkrankenhaus St. Marien gGmbH<br>Klinikum Neumarkt<br>Universitätsklinikum Erlangen<br>St. Theresien-Krankenhaus Nürnberg GmbH
2	Klinikum Nürnberg Nord<br>Klinikum Fürth<br>St. Anna Krankenhaus Sulzbach-Rosenberg<br>Klinik Hallerwiese (Nürnberg)<br>Kliniken d. Ldk. Neustadt a.d. Aisch - Bad Windsheim (Standort Neustadt a.d. Aisch)<br>Kreisklinik Roth<br>ANregiomed Klinikum Ansbach<br>310klinik GmbH (Nürnberg)*<br>Clinic Neuendettelsau*<br>Sana Klinik Pegnitz GmbH*<br>Stadtkrankenhaus Schwabach gGmbH*
3	Kliniken Altmühlfranken Weißenburg<br>Klinikum Forchheim<br>Sozialstiftung Bamberg<br>Krankenhaus Martha-Maria Nürnberg<br>Kliniken Dr. Erler gGmbH (Nürnberg)*<br>Kreiskrankenhaus St. Anna Höchstadt a. d. A.*
4	Steigerwaldklinik Burgebrach<br>Schön Klinik Nürnberg Fürth*
5	Krankenhäuser Nürnberger Land (KH Lauf)

Excluded due to low/missing number of cases:

Sana Klinik Nürnberg GmbH
Krankenhäuser Nürnberger Land (KH Altdorf)
Krankenhäuser Nürnberger Land (KH Hersbruck)
Cnopf'sche Kinderklinik (Klinik Hallerwiese Nürnberg)
Klinikum Nürnberg Süd

* Number of cases below average

Performance of local clinics

Clinics 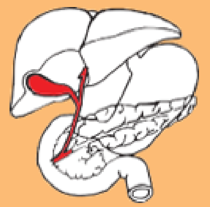	Medical quality information		number of cases[1]	patients' recommen-dation rate
	Number of unremarkable indicators from German external quality assurance (4 indicators in total)	Insurance claims data (routine data)		
310klinik GmbH (Nürnberg)	all unremarkable	average quality	-	95 %
ANregiomed Klinikum Ansbach	all unremarkable	average quality	ø	80 %
Clinic Neuendettelsau	all unremarkable	average quality	-	85 %
Cnopf'sche Kinderklinik (Klinik Hallerwiese Nbg.)	all unremarkable	number of cases too low to report	not specified	insuff. no. of ratings
Klinik Hallerwiese (Nürnberg)	all unremarkable	average quality	ø	88 %
Kliniken d. Ldkr. Neustadt a.d. A. Bad Windsheim[2]	all unremarkable	average quality	ø	82 %
Kliniken Dr. Erler gGmbH (Nürnberg)	3 out of 4 unremarkable	average quality	-	91 %
Kliniken Altmühlfranken Weißenburg	all unremarkable	below average	+	81 %
Klinikum Forchheim	all unremarkable	below average	+	81 %
Klinikum Fürth	all unremarkable	average quality	+	79 %
Klinikum Neumarkt	all unremarkable	above average	+	86 %
Klinikum Nürnberg Nord	3 out of 4 unremarkable	above average	+	80 %
Klinikum Nürnberg Süd	3 out of 4 unremarkable	number of cases too low to report	+	80 %
Krankenhaus Martha-Maria Nürnberg	all unremarkable	below average	ø	88 %
Krankenhäuser Nürnberger Land (KH Altdorf)	2 out of 4 unremarkable	number of cases too low to report	ø	84 %
Krankenhäuser Nürnberger Land (KH Hersbruck)	2 out of 4 unremarkable	number of cases too low to report	ø	84 %
Krankenhäuser Nürnberger Land (KH Lauf)	2 out of 4 unremarkable	below average	ø	84 %
Kreisklinik Roth	all unremarkable	average quality	ø	82 %
Kreiskrankenhaus St. Anna Höchstadt a. d. A.	all unremarkable	below average	-	76 %
Sana Klinik Pegnitz GmbH	all unremarkable	average quality	-	81 %
Sana Klinik Nürnberg GmbH	3 out of 4 unremarkable	average quality	-	83 %
Schön Klinik Nürnberg Fürth	3 out of 4 unremarkable	below average	-	88 %
Sozialstiftung Bamberg	3 out of 4 unremarkable	average quality	+	79 %
St. Anna Krankenhaus Sulzbach-Rosenberg	all unremarkable	average quality	ø	91 %
St. Theresien-Krankenhaus Nürnberg GmbH	all unremarkable	above average	ø	84 %
Stadtkrankenhaus Schwabach gGmbH	all unremarkable	average quality	-	76 %
Steigerwaldklinik Burgebrach	3 out of 4 unremarkable	below average	+	90 %
Universitätsklinikum Erlangen	all unremarkable	average quality	ø	86 %
Waldkrankenhaus St. Marien gGmbH	all unremarkable	average quality	+	89 %

[1] + number of cases above average, ø average number of cases, - number of cases below average, [2] Location: Neustadt a. d. Aisch

NZ INFOGRAFIK

Stationary treatment quality
in Nuremberg metropolitan area
Gallbladder Removal
2014

	Medical Quality Information							
	(1) German External Hospital Quality Assurance (esQS)							
Quality Indicator - abbreviation	220		50791		50824		51391	
Quality Indicator - serial number	Q1		Q2		Q3		Q4	
Quality Indicator - description	Occlusion or transection of the main bile duct		Ratio of the observed to the expected rate (O/E) of reinterventions due to complications		In-hospital mortality for low mortality risk		Ratio of the observed to the expected rate (O/E) of deaths	
Results (average in Germany)	0,12	SD	1,07	SD	0,12	SD	0,98	SD
Reference Range	sentinel event		0,00-2,35		sentinel event		0,00-3,24	
310klinik GmbH (Nürnberg)	0,00	R10	0,00	R10	0,00	R10	0,00	R10
ANregiomed Klinikum Ansbach	0,00	R10	0,22	R10	0,00	R10	0,43	R10
Clinic Neuendettelsau	0,00	R10	1,27	R10	0,00	R10	1,24	R10
Cnopf'sche Kinderklinik (Klinik Hallerwiese Nürnberg)		R10	0,00	R10		R10	0,00	R10
Klinik Hallerwiese (Nürnberg)	0,00	R10	0,93	R10	0,00	R10	0,00	R10
Kliniken des Landkreises Neustadt an der Aisch - Bad Windsheim	No data available							
Kliniken des Landkreises Neustadt an der Aisch - Bad Windsheim (Bad Windsheim)	0,00	R10	0,00	R10	0,00	R10	0,00	R10
Kliniken des Landkreises Neustadt an der Aisch - Bad Windsheim (Neustadt a.d. Aisch)		H20	0,73	R10	0,00	R10	0,67	R10
Kliniken Dr. Erler gGmbH (Nürnberg)	0,00	R10	1,72	R10	0,00	R10	0,00	R10
Klinikum Altmühlfranken Weißenburg	0,00	R10	0,95	R10	0,00	R10	2,52	R10
Klinikum Forchheim		H20	0,52	R10	0,00	R10	0,00	R10
Klinikum Fürth	0,00	R10	1,72	R10	0,00	R10	1,13	R10
Klinikum Neumarkt		H20	1,13	R10	0,00	R10	1,82	R10
Klinikum Nürnberg	No data available							
Klinikum Nürnberg (Nord)		H20	0,95	R10		U32	1,10	R10
Klinikum Nürnberg (Süd)	0,00 [13]	R10	2,15 [13]	R10	0,00 [13]	R10	0,00 [13]	R10
Krankenhaus Martha-Maria Nürnberg	0,00	R10	2,05	R10	0,00	R10	0,00	R10
Krankenhäuser Nürnberger Land GmbH	No data available							
Krankenhäuser Nürnberger Land (Krankenhaus Altdorf)	No data available							
Krankenhäuser Nürnberger Land (Krankenhaus Hersbruck)	No data available							
Krankenhäuser Nürnberger Land GmbH (Krankenhaus Lauf)		H20	3,47	H20	0,00	R10	0,55	R10
Kreisklinik Roth	0,00	R10	0,42	R10	0,00	R10	0,58	R10
Kreiskrankenhaus St.Anna Höchstadt a.d. Aisch	0,00	R10	1,63	R10	0,00	R10	0,00	R10
Sana Klinik Pegnitz GmbH	0,00	R10	2,30	R10	0,00	R10	0,00	R10
Sana-Klinik Nürnberg GmbH	0,00	R10	0,00	R10	0,00	R10	0,00	R10
Schön Klinik Nürnberg Fürth	0,00	R10	0,00	R10	0,00	R10	0,00	R10
Sozialstiftung Bamberg	0,00	R10	0,56	R10	0,00	R10	1,07	R10
St. Anna Krankenhaus Sulzbach-Rosenberg	0,00	R10	1,16	R10	0,00	R10	1,27	R10
St. Theresien-Krankenhaus Nürnberg gGmbH	0,00	R10	0,99	R10	0,00	R10	0,00	R10
Stadtkrankenhaus Schwabach gGmbH		U32	0,72	R10	0,00	R10	1,77	R10
Steigerwaldklinik Burgebrach	0,00	R10	1,28	R10	0,00	R10	0,00	R10
Universitätsklinikum Erlangen	0,00	R10	0,80	R10		H20	3,04	R10
Waldkrankenhaus St. Marien gGmbH	0,00	R10	0,41	R10	0,00	R10	0,00	R10

Stationary treatment quality
in Nuremberg metropolitan area

Gallbladder Removal

2014

	Medical Quality Information														
	(2) Insurance Claims Data (Routine Data)														
Quality Indicator - abbreviation	Overall Result			Q1			Q2			Q3					
Quality Indicator - serial number															
Quality Indicator - description	"AOK-Lebensbäume" (insurance internal rating system)			Transfusion/bleeding during hospitalization			Other complications after the procedure			Mortality within 90 days after the procedure			(3) Number of Cases (German External Quality Assurance)	(4) Patients' recommendation rate
Results (average in Germany)															82%
Reference Range	quantity	value	CI	assessment	value	CI	assessment	value	CI	assessment	value	CI			
310klinik GmbH (Nürnberg)	2	1,70	(0,2 - 3,1)	average	1,60	(0,0 - 4,1)	average	1,50	(0,0 - 3,1)	average	0,00	(0,0 - 6,0)	74	96%
ANregiomed Klinikum Ansbach	2	0,90	(0,5 - 1,4)	average	0,80	(0,2 - 1,5)	average	0,90	(0,3 - 1,5)	average	1,10	(0,1 - 2,1)	185	80%
Clinic Neuendettelsau	2	0,80	(0,2 - 1,4)	average	1,30	(0,4 - 2,2)	above average	0,50	(0,0 - 1,3)	above average	0,00	(0,0 - 1,6)	101	86%
Cnopf'sche Kinderklinik (Klinik Hallerwiese Nürnberg)	no. of cases too low			no. of cases too low			no. of cases too low			no. of cases too low			no data available	insufficient no. of ratings
Klinik Hallerwiese (Nürnberg)	2	0,50	(0,0 - 1,2)	above average	0,00	(0,0 - 1,3)	average	0,70	(0,0 - 1,5)	average	0,00	(0,0 - 3,5)	197	88%
Kliniken des Landkreises Neustadt an der Aisch - Bad Windsheim	no data available												183	not specified
Kliniken des Landkreises Neustadt an der Aisch - Bad Windsheim (Bad Windsheim)	no. of cases too low			no. of cases too low			no. of cases too low			no. of cases too low			27	82%
Kliniken des Landkreises Neustadt an der Aisch - Bad Windsheim (Neustadt a.d. Aisch)	2	1,00	(0,7 - 1,4)	below average	1,40	(0,7 - 2,0)	average	0,90	(0,4 - 1,4)	average	0,90	(0,0 - 1,9)	156	82%
Kliniken Dr. Erler gGmbH (Nürnberg)	2	1,60	(0,4 - 2,8)	average	3,40	(1,3 - 5,5)	average	1,60	(0,2 - 3,0)	average	0,00	(0,0 - 5,5)	124	91%
Klinikum Altmühlfranken Weißenburg	1	1,20	(0,8 - 1,5)	below average	1,60	(1,0 - 2,2)	average	1,00	(0,5 - 1,4)	above average	0,60	(0,0 - 1,6)	229	81%
Klinikum Forchheim	1	1,30	(0,9 - 1,8)	average	1,30	(0,5 - 2,0)	below average	1,60	(1,0 - 2,3)	average	1,10	(0,0 - 2,5)	165	81%
Klinikum Fürth	2	0,90	(0,5 - 1,3)	average	1,20	(0,5-1,9)	above average	0,70	(0,2 - 1,3)	above average	0,00	(0,0 - 1,4)	274	79%
Klinikum Neumarkt	3	0,90	(0,5 - 1,2)	above average	0,40	(0,0 - 1,0)	average	1,10	(0,6 - 1,6)	average	0,90	(0,0 - 1,9)	306	85%
Klinikum Nürnberg	no data available												589	not specified
Klinikum Nürnberg (Nord)	3	0,90	(0,7 - 1,0)	above average	0,40	(0,1 - 0,6)	average	1,00	(0,7 - 1,2)	average	0,90	(0,4 - 1,3)	577	79%
Klinikum Nürnberg (Süd)	no. of cases too low			no. of cases too low			no. of cases too low			no. of cases too low			12 [14]	79%
Krankenhaus Martha-Maria Nürnberg	1	1,40	(0,8 - 2,0)	average	1,50	(0,5 - 2,6)	average	1,30	(0,6 - 2,1)	average	0,70	(0,0 - 2,0)	251	89%
Krankenhäuser Nürnberger Land GmbH	no data available												191	not specified
Krankenhäuser Nürnberger Land (Krankenhaus Altdorf)	no. of cases too low			no. of cases too low			no. of cases too low			no. of cases too low			no data available	84%
Krankenhäuser Nürnberger Land (Krankenhaus Hersbruck)	no. of cases too low			no. of cases too low			no. of cases too low			no. of cases too low			no data available	84%
Krankenhäuser Nürnberger Land GmbH (Krankenhaus Lauf)	1	1,20	(0,9 - 1,6)	average	1,00	(0,5 - 1,6)	below average	1,40	(0,9 - 1,8)	above average	0,50	(0,0 - 1,3)	no data available	84%
Kreisklinik Roth	2	0,80	(0,1 - 1,5)	average	1,00	(0,0 - 1,9)	average	0,60	(0,0 - 1,6)	average	0,80	(0,0 - 2,4)	210	82%
Kreiskrankenhaus St.Anna Höchstadt a.d. Aisch	1	2,60	(1,5 - 3,7)	average	2,20	(0,2 - 4,2)	average	2,30	(0,9 - 3,8)	average	3,00	(0,0 - 6,3)	25	insufficient no. of ratings
Sana Klinik Pegnitz GmbH	2	0,60	(0,0 - 1,4)	average	0,90	(0,0 - 2,2)	average	0,50	(0,0 - 1,4)	average	1,40	(0,0 - 3,6)	74	80%
Sana-Klinik Nürnberg GmbH	2	1,00	(0,0 - 2,9)	average	3,00	(0,0 - 6,4)	average	0,00	(0,0 - 2,3)	average	0,00	(0,0 - 8,5)	17	83%
Schön Klinik Nürnberg Fürth	1	6,70	(5,3 - 8,0)	below average	6,70	(4,4 - 8,9)	below average	7,50	(5,8 - 9,2)	average	0,00	(0,0 - 5,0)	45	88%
Sozialstiftung Bamberg	2	1,00	(0,7 - 1,3)	above average	0,80	(0,3 - 1,3)	average	1,00	(0,7 - 1,4)	above average	0,60	(0,0 - 1,3)	214	79%
St. Anna Krankenhaus Sulzbach-Rosenberg	2	0,80	(0,1 - 1,5)	average	1,20	(0,1 - 2,3)	average	0,80	(0,0 - 1,7)	average	0,00	(0,0 - 1,9)	140	91%
St. Theresien-Krankenhaus Nürnberg gGmbH	3	0,60	(0,1 - 1,1)	average	1,00	(0,2 - 1,8)	average	0,70	(0,0 - 1,3)	average	1,70	(0,3 - 3,1)	202	85%
Stadtkrankenhaus Schwabach gGmbH	2	0,80	(0,2 - 1,4)	average	0,60	(0,0 - 1,6)	average	1,20	(0,4 - 2,1)	average	0,90	(0,0 - 2,6)	108	76%
Steigerwaldklinik Burgebrach	1	1,30	(0,8 - 1,8)	average	1,30	(0,4 - 2,2)	average	1,00	(0,3 - 1,7)	below average	3,70	(2,2 - 5,1)	115	90%
Universitätsklinikum Erlangen	3	0,60	(0,2 - 1,0)	average	0,80	(0,1 - 1,5)	above average	0,40	(0,0 - 0,9)	average	1,30	(0,1 - 2,5)	205	87%
Waldkrankenhaus St. Marien gGmbH	3	0,30	(0,0 - 0,8)	average	0,80	(0,0 - 1,8)	above average	0,00	(0,0 - 0,7)	average	0,00	(0,0 - 2,0)	223	89%

Stationary treatment quality
in Nuremberg metropolitan area

Gallbladder Removal

2014

Legend

A40	Erroneous documentation will be confirmed (data validation)	Ranked as qualitatively discrepant after Structured Dialogue
A41	Notices on structural or process deficiencies	
A42	No (sufficiently explanatory) reasons known for the computational discrepancy	
A99	Other (explained in the comment)	
D50	Incomplete or erroneous documentation	Evaluation not possible due to improper documentation
D51	Software problems caused erroneous documentation	
D99	Other (explained in the comments)	
H20	Request on internal quality management to establish the analysis of calculated abnormalities	Remarkable result concerning calculations was pointed out to the insitution
H99	Other (explained in the commentary)	
U30	Correct documentation will be confirmed (data validation)	Quality assessment is unremarkable after structured dialog
U31	Special clinical situation	
U32	The deviating result explained by isolated cases	
U33	No indication for medical quality faults (isolated documentation problems)	
U99	Other (explained in the comments)	
R10	Result computationally non-discrepant, therefore no Structured Dialogue required	Result within reference range
N01	Quality indicator without a result because no corresponding cases occurred	Evaluation not planned
N02	Reference range not defined for this indicator	
N99	Other (explained in the comments)	

Note:
Not highlighted cells have not been assessed as qualitatively unremarkable.
Cells marked in yellow have been assessed as qualitatively unremarkable.
Cells marked in grey have not been considered in the assessement.

CI	Confidence Interval
SD	Structured Dialog
UE	Unfavourable single-event
1	Presumably data show inconsistencies. It is presumed that Bad Windsheim is performing this service.
2	Insurance claims data are referring to Waldkrankenhaus St. Marien gGmbH as a whole.
3	May refer to more than one hospital location.
4	Refers to all hospital locations.
5	Incorrect documentation in single-events. Analysis of cases ensued. No indication for medical quality faults.
6	The herein analyzed treatment was performed at Klinikum Nürnberg Süd. For single-events, patients are transferred to Klinikum Nürnberg Nord, e.g. due to comorbidities. Based on legal regulations, these patients must be shown here. Nevertheless the
7	The main speciality department is located at Klinikum Süd. Treatement was executed there.
8	In the meanwhile a new cardiac catheterization lab has been commissioned.
9	Due to the commissioning of two new cardiac catheterization labs, the dosis protocols had to be ad usted.
10	A significantly higher number of pressure wires (fractional coronary flow reserve) was performed to evaluate haemodynamical relevance of intermediary stenosis. Additionally a stent-enhancement-procedure was introduced.
11	No quality faults affirmed after case analysis.
12	Result is not rateable qualitatively.
13	The herein analyzed treatment was performed at Klinikum Nürnberg Nord. For single-events, patients are transferred to Klinikum Nürnberg Süd, e.g. due to comorbidities. Based on legal regulations, these patients must be shown here. Nevertheless the
14	The main speciality department is located at Klinikum Nord. Treatement was executed there.
