# Supplementary material for: Public release of hospital quality data for referral practices in Germany: results from a cluster-randomised controlled trial
Source: Health Econ Rev. 2017 Sep 26;7:33. doi: 10.1186/s13561-017-0171-5 (PMC5615085; doi:10.1186/s13561-017-0171-5)
Supplement: Supplementary file 2 — The survey instrument. (DOCX 623 kb) [file 13561_2017_171_MOESM2_ESM.docx]

***Part I: Nuremberg hospital quality reporting system (NHQRS)***

On 16**^th^** January 2016, the results for the clinical procedure “**gallbladder removal**“ were published.

| 1. Have you ever heard of NHQRS? | | |
| --- | --- | --- |
|  | I am not familiar with NHQRS. *[🡪 Skip to Part II]* | 🔾 |
|  | I have heard of NHQRS but I did not read the newspaper article. | 🔾 |
|  | I scanned the newspaper article. | 🔾 |
|  | I took a closer look at NHQRS. | 🔾 |

| 2. | How did you become aware of NHQRS? [multiple choices possible] | | | |
| --- | --- | --- | --- | --- |
|  | Contact with patients | 🔾 | Internet | 🔾 |
|  | Peers | 🔾 | Public reporting | 🔾 |
|  | I am a reader of “Nürnberger Zeitung”. | 🔾 | Lecture | 🔾 |
|  | Others:  🖉  ………………………………………………………………………………………………………………………. | | | |

| 3. | Did the ranking influence you in a way that you have decided… |  |  |
| --- | --- | --- | --- |
|  |  | Yes | No |
|  | … to send patients to a specific hospital? | 🔾 | 🔾 |
|  | … not to send patients to a specific hospital? | 🔾 | 🔾 |

***Part II: Different presentation formats***

The results are published in three different presentation formats.

1) **Overall hospital ranking** (hospitals were assigned to three resp. five ranking groups)

2) **Alphabetical overview** (shows quality information, number of cases and the patients’ recommendation rate of all hospitals)

3) **Detailed presentation format** (shows detailed information on a quality-indicator based level)

The **overall hospital ranking** and the **alphabetical overview** were published in the newspaper, the **detailed presentation format** can be accessed online. Due to lack of space, all three figures can be viewed in the additional material. In the following sections, every presentation format is displayed and we kindly ask you to answer some questions about the presentation formats.

***Presentation format 1: Overall ranking***

| 4. | How **helpful** do you perceive this presentation format for patient counseling in everyday practice? | | | | | | |
| --- | --- | --- | --- | --- | --- | --- | --- |
|  | Not helfpul at all | 🔾 | 🔾 | 🔾 | 🔾 | 🔾 | Very helpful |


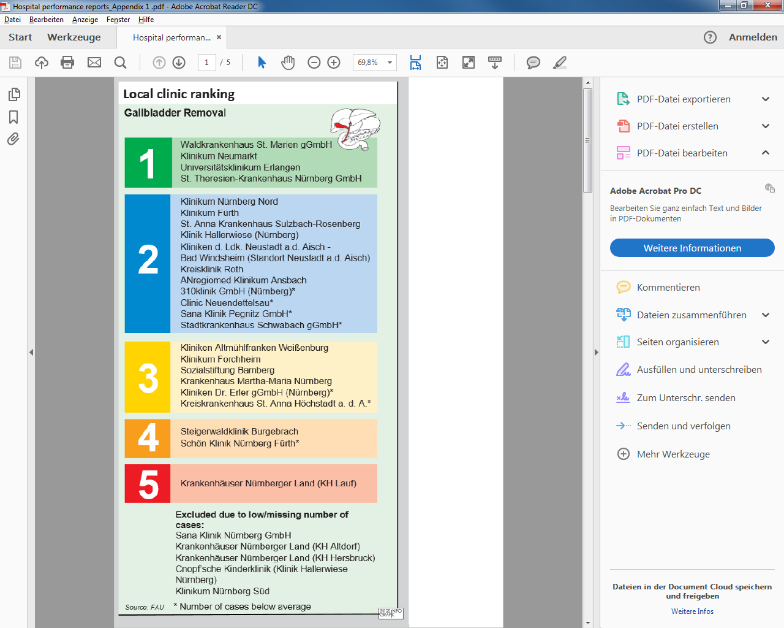


| 5. | How likely is it, that this presentation format will **influence your referral patterns**? | | | | | | |
| --- | --- | --- | --- | --- | --- | --- | --- |
|  | Not likely at all | 🔾 | 🔾 | 🔾 | 🔾 | 🔾 | Very likely |

| 6. | Please answer the following questions about this **presentation format.** | | | |
| --- | --- | --- | --- | --- |
|  |  | Yes | No | I don´t know |
|  | Do you think that this aggregated presentation format is trustworthy? | 🔾 | 🔾 | 🔾 |
|  | Do you think it makes sense to present different performance groups? | 🔾 | 🔾 | 🔾 |
|  | Would you recommend to present only the best hospitals? | 🔾 | 🔾 | 🔾 |
|  | Do you think low performing hospitals should be presented as well? | 🔾 | 🔾 | 🔾 |

***Presentation format 2: Alphabetical overview***

| 7. | How **helpful** do you perceive this presentation format for patient counseling in everyday practice? | | | | | | |
| --- | --- | --- | --- | --- | --- | --- | --- |
|  | Not helpful at all | 🔾 | 🔾 | 🔾 | 🔾 | 🔾 | Very helpful |


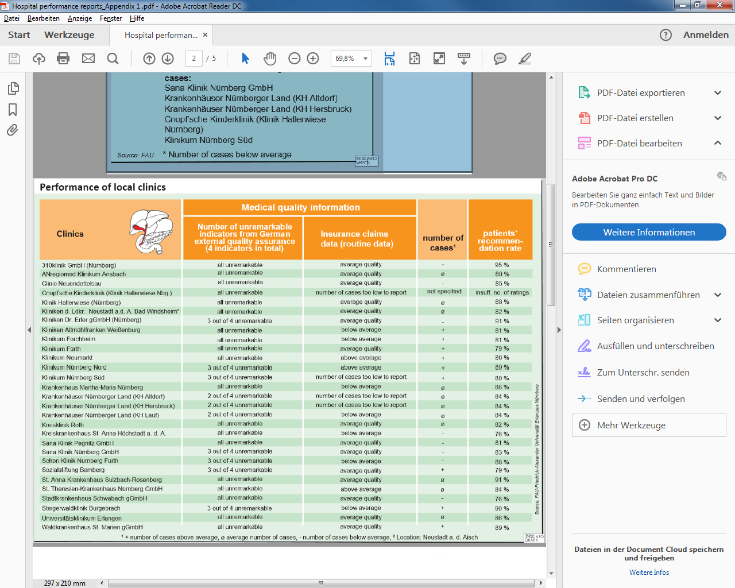


| 8. | How likely is it, that this presentation format will **influence your referral patterns**? | | | | | | |
| --- | --- | --- | --- | --- | --- | --- | --- |
|  | Not likely at all | 🔾 | 🔾 | 🔾 | 🔾 | 🔾 | Very likely |

| 9. How relevant is the following information for your referral decisions? | | | | | | |
| --- | --- | --- | --- | --- | --- | --- |
|  |  | No relevant at all |  |  |  | Highly relevant |
|  | Column 1: German external quality assurance | 🔾 | 🔾 | 🔾 | 🔾 | 🔾 |
|  | Column 2: Insurance claims data (routine data) | 🔾 | 🔾 | 🔾 | 🔾 | 🔾 |
|  | Column 3: Number of cases | 🔾 | 🔾 | 🔾 | 🔾 | 🔾 |
|  | Column 4: Patients‘ recommendation result | 🔾 | 🔾 | 🔾 | 🔾 | 🔾 |

***Presentation format 3: Detailed presentation format***

| 10. | How **helpful** do you perceive this presentation format for patient counseling in everyday practice? | | | | | | |
| --- | --- | --- | --- | --- | --- | --- | --- |
|  | Not helpful at all | 🔾 | 🔾 | 🔾 | 🔾 | 🔾 | Very helpful |


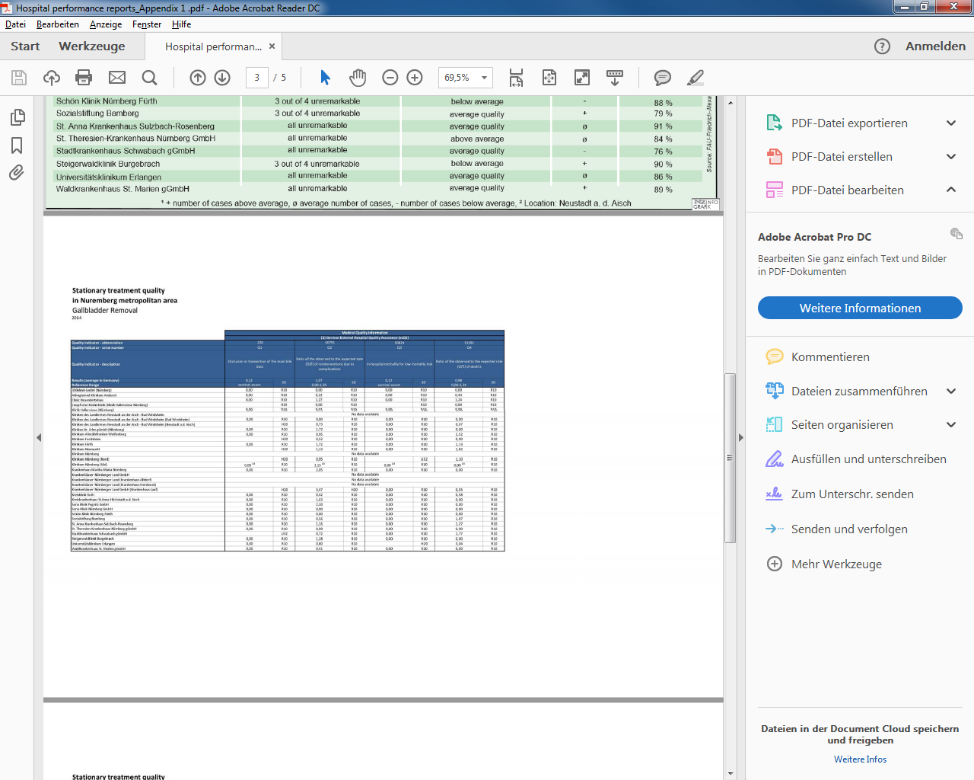


| 11. | How likely is it, that this presentation format will **influence your referral patterns**? | | | | | | |
| --- | --- | --- | --- | --- | --- | --- | --- |
|  | Not likely at all | 🔾 | 🔾 | 🔾 | 🔾 | 🔾 | Very likely |

| 12. | Please answer the following questions about **this presentation format.** | | | |
| --- | --- | --- | --- | --- |
|  |  | Yes | No | I don’t know |
|  | Have you downloaded the detailed information before starting this survey? | 🔾 | 🔾 | 🔾 |
|  | Do you think this level of detail is necessary to get enough information about the quality of a hospital? | 🔾 | 🔾 | 🔾 |
|  | Do you think the information is prepared in a comprehensible way? | 🔾 | 🔾 | 🔾 |

***Part IV: Final questions***

| 13. How would you assess the NHQRS in general? | | | | | | | |
| --- | --- | --- | --- | --- | --- | --- | --- |
|  | Not trustworthy at all | 🔾 | 🔾 | 🔾 | 🔾 | 🔾 | Very trustworthy |
|  | Not helpful for patient counseling at all | 🔾 | 🔾 | 🔾 | 🔾 | 🔾 | Very helpful for patient counseling |
|  | Not credible at all | 🔾 | 🔾 | 🔾 | 🔾 | 🔾 | Very credible |
|  | Not meaningful at all | 🔾 | 🔾 | 🔾 | 🔾 | 🔾 | Very meaningful |

| 14. How do you evaluate the NHQRS by using German school grades? | | | | | | | | |
| --- | --- | --- | --- | --- | --- | --- | --- | --- |
|  | Grade 1 (very good) | 🔾 | 🔾 | 🔾 | 🔾 | 🔾 | 🔾 | Grade 6 (insufficient) |

| 15. Do the presented quality results reflect your own experiences? | | |  | 16. Will you consider NHQRS for future referral decisions? | | |  |
| --- | --- | --- | --- | --- | --- | --- | --- |
|  | Yes | 🔾 |  | | Yes | 🔾 |  |
|  | No | 🔾 |  |  | No | 🔾 |  |
|  | Partly | 🔾 |  |  | I don’t know | 🔾 |  |
|  | I don’t know | 🔾 |  | |  |  |  |

| 17. | Generally speaking, how necessary do you consider initiatives to increase the transparency about the quality of hospitals? (e.g. NHQRS) | | | | | | |
| --- | --- | --- | --- | --- | --- | --- | --- |
|  | Not necessary at all | 🔾 | 🔾 | 🔾 | 🔾 | 🔾 | Very necessary |

| 18. Do you think that NHQRS should be repeated periodically? | Yes | No | I don’t know |
| --- | --- | --- | --- |
|  | 🔾 | 🔾 | 🔾 |

| 19. | Finally, we would like to know your opinion concerning **criticism** and **improvement suggestions**. |
| --- | --- |
|  | 🖉 |

***Part V: General information about your person***

| 20. Please indicate your gender. | | | | 21. Please indicate your year of birth [e.g., 1984]. | | |  | 22. Do you perform the procedure “gallbladder removal“ yourself? | | | |
| --- | --- | --- | --- | --- | --- | --- | --- | --- | --- | --- | --- |
|  | Male | 🔾 |  | | 🖉………………………….... |  |  | | Yes | 🔾 |  |
|  | Female | 🔾 |  |  |  |  |  | | No | 🔾 |  |

| 23. | In what kind of practice do you work? | | | |  | 24. Which of the following medical trainings have you completed? | | | |
| --- | --- | --- | --- | --- | --- | --- | --- | --- | --- |
|  | Single practice | 🔾 | Medical care center | 🔾 |  | General practitioner | 🔾 | Internist (primary care) | 🔾 |
|  | “Praxisgemeinschaft” (Joint practice by only sharing equipment etc.) | 🔾 | Group practice | 🔾 |  | Medical practitioner | 🔾 | Internist (specialist) | 🔾 |
|  | Others: 🖉 ……………………………………………………… | | | |  |  | | | |
